# Genomic traits of multidrug resistant enterotoxigenic *Escherichia coli* isolates from diarrheic pigs

**DOI:** 10.3389/fmicb.2023.1244026

**Published:** 2023-08-03

**Authors:** Jiameng Hu, Junlin Li, Xiaobo Huang, Jing Xia, Min Cui, Yong Huang, Yiping Wen, Yue Xie, Qin Zhao, Sanjie Cao, Likou Zou, Xinfeng Han

**Affiliations:** ^1^College of Veterinary Medicine, Sichuan Agricultural University, Chengdu, China; ^2^Engineering Research Center of Southwest Animal Disease Prevention and Control Technology, Ministry of Education of China, Chengdu, China; ^3^Key Laboratory of Animal Disease and Human Health of Sichuan Province, Chengdu, China; ^4^College of Resources, Sichuan Agricultural University, Chengdu, China

**Keywords:** Enterotoxigenic *Escherichia coli*, whole-genome sequencing, antimicrobial resistance, genomic characterization, virulence factor, pig

## Abstract

Diarrhea caused by enterotoxigenic *Escherichia coli* (ETEC) infections poses a significant challenge in global pig farming. To address this issue, the study was conducted to identify and characterize 19 ETEC isolates from fecal samples of diarrheic pigs sourced from large-scale farms in Sichuan Province, China. Whole-genome sequencing and bioinformatic analysis were utilized for identification and characterization. The isolates exhibited substantial resistance to cefotaxime, ceftriaxone, chloramphenicol, ciprofloxacin, gentamicin, ampicillin, tetracycline, florfenicol, and sulfadiazine, but were highly susceptible to amikacin, imipenem, and cefoxitin. Genetic diversity among the isolates was observed, with serotypes O22:H10, O163orOX21:H4, and O105:H8 being dominant. Further analysis revealed 53 resistance genes and 13 categories of 195 virulence factors. Of concern was the presence of *tet*(X4) in some isolates, indicating potential public health risks. The ETEC isolates demonstrated the ability to produce either heat-stable enterotoxin (ST) alone or both heat-labile enterotoxin (LT) and ST simultaneously, involving various virulence genes. Notably, STa were linked to human disease. Additionally, the presence of 4 hybrid ETEC/STEC isolates harboring Shiga-like toxin-related virulence factors, namely *stx2a*, *stx2b*, and *stx2e-ONT-2771*, was identified. IncF plasmids carrying multiple antimicrobial resistance genes were prevalent, and a hybrid ETEC/STEC plasmid was detected, highlighting the role of plasmids in hybrid pathotype emergence. These findings emphasized the multidrug resistance and pathogenicity of porcine-origin ETEC strains and the potential risk of epidemics through horizontal transmission of drug resistance, which is crucial for effective control strategies and interventions to mitigate the impact on animal and human health.

## 1. Introduction

Enterotoxigenic *Escherichia coli* (ETEC) is a significant cause of diarrhea in both humans and animals (Francis, 2002; Esfandiari et al., 2017). Post-weaning diarrhea (PWD) typically manifests within 2 weeks following weaning, and is characterized by clinical signs including diarrhea, dehydration, and mortality. ETEC is the most frequently isolated pathogen from PWD outbreaks (Fairbrother et al., 2005), and is distinguished by its production of fimbriae and enterotoxins. It can be spread through feces, water, and food. Due to its high morbidity and mortality rates, it significantly affects piglet performance and causes significant economic losses in pig industry globally (Luppi, 2017; García-Meniño et al., 2021).

Although antimicrobial agents may provide temporary relief and help control the disease burden in piglets, their prophylactic and therapeutic use may lead to the establishment of antimicrobial-resistant bacteria in the gut microbiota, which poses a threat to both environment and public health. PWD is one of the most common reasons for the use of antimicrobial agents in the pig industry worldwide and contributes significantly to the problem of antimicrobial resistance (AMR) development (Luppi, 2017; Rhouma et al., 2017; Luise et al., 2019; Laird et al., 2021). Chromosomes and plasmids play crucial roles in the field of public health, particularly when it comes to antibiotic resistance and the presence of virulence genes. Genes responsible for virulence in *E. coli* strains can frequently be transmitted between them, residing either on chromosomes, plasmids, or phages. The emergence of antibiotic-resistant bacteria can be attributed to genetic mutations within chromosomes or the acquisition of genetic material, such as resistance plasmids (Bakkeren et al., 2019). AMR genes such as *mcr*, *tet*(X4), ESBL genes like *bla*_CTX-M_ and *bla*_TEM_, and carbapenem-resistant *Enterobacteriaceae* (CRE) associated genes like *bla*_KPC_ and *bla*_NDM_ present substantial threats to public health (Faccone et al., 2019; Nguyet et al., 2022; Wang et al., 2022; Zhang et al., 2022).

ETEC from pigs produce two enterotoxins, namely, heat-labile enterotoxin (LT) and heat-stable enterotoxin (STa and STb), both of which are encoded by plasmids (Johnson and Nolan, 2009). There are distinct disparities in the LT and ST enterotoxins produced by porcine and human ETEC strains. Despite a high degree of nucleotide sequence similarity between the *eltAB* genes (pLT and hLT) and the *estA* genes (pSTa and hSTa), the absence of hLT and hSTa toxins in porcine ETEC strains causing diarrhea is noteworthy. Additionally, there are variations in epitopes and antigenicities between the toxins of porcine and human origin (Zhang et al., 2008). ETEC pathotypes in pigs are characterized by specific fimbrial adhesins that allow bacterial colonization of the gut mucosal surface. ETEC uses fimbriae to adhere to specific receptors on the target host cells and subsequently colonizes piglet small intestinal epithelial cells (Smith and Linggood, 1971). Once established in the intestine, ETEC secretes enterotoxins that disrupt fluid homeostasis, resulting in watery diarrhea by increasing secretion and inhibiting absorption in the intestine (Dubreuil et al., 2016; Sun and Kim, 2017). ETEC strains causing porcine diarrhea include five different fimbrial subtypes, namely K88 (F4), K99 (F5), F41, F18, and 987P (F6) (Isaacson, 1977; Isaacson and Richter, 1981; De Graaf and Roorda, 1982; Bakker et al., 1991; Nagy et al., 1997). Compared with ETEC strains that infect humans, the predominant adhesins found in ETEC strains isolated from pigs with PWD are fimbriae F4 and F18 (García et al., 2020). In contrast, fimbriae F5, F6 and F41 are typically associated with ETEC-induced neonatal diarrhea in piglets, and have little correlation with PWD (Frydendahl, 2002; Zhang et al., 2007). The fimbriae of ETEC have both immunogenic and antigenic properties and are the primary target for developing vaccines against this pathogen (Ntakiyisumba et al., 2022). However, due to the prevalence of mixed infections and the diversity of ETEC pilus types, it remains challenging to determine the specific types of pili carried by infected individuals, which presents a significant obstacle to effective disease prevention and control.

Shiga toxin-producing *Escherichia coli* (STEC) strains are globally recognized as significant causes of diarrhea in both humans and animals (Lee et al., 2023). These strains are known for their ability to produce one or two distinct types of Shiga toxin (Stx), which serve as the primary virulence factor. Shiga toxins, classified as Stx1 and Stx2, play a crucial role in the pathogenicity of STEC (Melton-Celsa, 2014). Previous studies have shown that ETEC strains, capable of producing Stx toxins, are often classified as ETEC/STEC hybrid strains (Fairbrother et al., 2005).

Whole-genome sequencing (WGS) has become increasingly accessible to clinical and microbiological laboratories in recent decades due to a decrease in cost. This technology is a powerful tool for analyzing bacterial genomes rapidly (Kwong et al., 2015). In this study, WGS technology was employed in conjunction with sophisticated bioinformatics analysis to comprehensively investigate a multitude of pivotal aspects pertaining to ETEC isolates. The investigative scope encompassed an in-depth exploration of serotype prevalence, antimicrobial resistance phenotypes, antimicrobial resistance genes (ARGs), virulence factors (VFs), and plasmid incompatibility types. Furthermore, a comparative analysis was conducted to elucidate the phylogenetic relationship between ETEC strains under investigation and ETEC strains originating from diverse geographic regions worldwide.

To enhance understanding of porcine ETEC, it is crucial to monitor its entire genome. However, the current knowledge is hindered by some gaps and limitations. The gaps include limited knowledge of genomic diversity, detection of different serotypes in different regions, geographical representation in public databases, and comparative analysis with human ETEC strains. This hampers the ability to accurately assess global genetic diversity, represent various lineages and regional variations, and may result in a geographic bias in available genomes. The primary objective of this study was to achieve a thorough genomic characterization of ETEC strains isolated in China, thus contributing reference data that can significantly inform the development and implementation of targeted disease control strategies.

## 2. Materials and methods

### 2.1. Bacterial isolation and identification of *Escherichia coli* from fecal samples

Samples of diarrheal feces were collected using swabs from large-scale pig farms in Sichuan Province between 2020 and 2021, which is recognized as one of the largest pig-breeding districts in China. Samples were obtained from over 10 large-scale pig farms located in various regions of Sichuan Province, China, including Wansheng Town (Chengdu City), Dayi County (Chengdu City), Hongya County (Meishan City), and Qinjia Town (Ya’an City). The collected samples were then transferred to 10 mL centrifuge tubes filled with sterile PBS. Each sample was assigned a unique number and carefully stored in insulated foam boxes along ice packs. Within 24 h, the samples were transported back to the laboratory under low-temperature conditions to facilitate further processing. Subsequently, the collected swabs were subjected to incubation on MacConkey agar, and the presence of *Escherichia coli* was confirmed by consulting the Bergey’s Manual of Bacterial Identification (Holt et al., 1994). The confirmation involved conducting multiple biochemical assays, including gram staining, oxidase and catalase tests, and metabolic assessments on different media like Methyl Red and Vogues-Proskauer broth (MR-VP), Triple Sugar Iron (TSI), Sulfide indole motility (SIM), Simmons’ Citrate, and Eosin Methylene Blue (EMB) agar. Colonies exhibiting characteristic morphology were identified as *Escherichia coli* (*E. coli*). The extraction of genomic DNA from *E. coli* isolates was performed using the TIANamp Bacteria DNA Kit (Tiangen Biotech) for further identification.

### 2.2. Molecular identification of ETEC

In order to identify ETEC out of the *E. coli* colonies, specific primers targeting LT and ST sequences were designed according to previous report (Table 1) (Zhang et al., 2007). The PCR reaction mixture contained 12.5 μL of Premix Taq™, 1 μL of each upstream and downstream primer, 2 μL of extracted DNA samples, and 8.5 μL of ddH_2_O, for a total volume of 25 μL. The PCR amplification program consisted of an initial denaturation at 94°C for 5 min, followed by 30 cycles of denaturation at 95°C for 30 s, annealing for 30 s, and extension at 72 s°C for 30 s. The PCR products were separated on a 1% agarose gel and visualized using a gel imager. The identified ETEC strains were preserved at −20°C in Luria-Bertani (LB) broth supplemented with 20% glycerol.

**Table 1:** PCR primer sequences for identification of ETEC.

Primer	Sequence (5′–3′)	Base number	Product size	Annealing temperature
LT (F)	CACGCGAGAGGAACACAAAC	20	399 bp	57.5°C
LT (R)	TGTAACCATCCTCTGCCGGA	20		
STa (F)	AAAAGCTAATGTTGGCAAT	19	196 bp	49.1°C
STa (R)	GCAGGATTACAACAAAGTTCA	21		
STb (F)	GCTACAAATGCCTATGCATCTACACA	26	156 bp	59.6°C
STb (R)	CATGCTCCAGCAGTACCATCTCTAAC	26		

### 2.3. Antimicrobial susceptibility testing

To determine the antimicrobial susceptibility profile of ETEC isolates, the micro-broth dilution method was utilized following Clinical and Laboratory Standards Institute (CLSI) guidelines (CLSI, 2021). A variety of antimicrobial agents, including amikacin (AMK), gentamicin (GEN), streptomycin (STR), cefoxitin (FOX), cefotaxime (CTX), ceftriaxone (CRO), ampicillin (AMP), imipenem (IPM), polymyxin B (PMB), tetracycline (TET), chloramphenicol (CHL), florfenicol (FFC), ciprofloxacin (CIP), nalidixic acid (NAL), and sulfasalazine (SSZ), were tested, and their minimum inhibitory concentration (MIC) values were recorded. The MIC values were compared with the latest CLSI standards, with *E. coli* ATCC25919 serving as the quality control strain. The results were categorized as susceptible (S), intermediate (I) or resistant (R). Isolates showing resistance to antimicrobial agents from three or more classes were defined as multidrug resistant (MDR).

### 2.4. Whole-genome sequencing and bioinformatic analysis

Genomic DNA was sent to Shanghai Ling’en Biotechnology Co. Ltd. (Shanghai, China) for sequencing, and its quality and concentration were evaluated using a NanoDrop2000 system and a Qubit 4 Fluorometer. The NEBNext Ultra^™^ II DNA Library Prep Kit was utilized for constructing libraries using qualified DNA. These libraries were then sequenced on an Illumina NovaSeq 6000 platform utilizing a paired-end 150 bp sequencing by synthesis protocol. To ensure data quality, low-quality raw reads were excluded based on predefined criteria. Data assembly followed the removal of adapter contamination and data filtering using AdapterRemoval and SOAPec. High-quality reads were assembled into genome contigs using SPAdesv 3.13.0.

To predict and analyze antibiotic resistance, the Comprehensive Antibiotic Resistance Gene Database (CARD)^1^ and ResFinder 4.1^2^ were employed. For the prediction and analysis of virulence genes, the Virulence Factors of Pathogenic Bacteria Database (VFDB)^3^ and VirulenceFinder 2.0^4^ were utilized. During the search process in ResFinder 4.1, the thresholds for gene identity and minimum length were established at 90% and 60%, respectively. Similarly, the VirulenceFinder database employed thresholds of 95% for gene identity and 60% for alignment coverage during the analysis process. The PlasmidFinder 2.1 online analysis platform^5^ was utilized to determine plasmid incompatibility types (Carattoli et al., 2014). The PlasmidFinder database utilized a minimum alignment coverage of ≥60% and required an identity percentage of ≥95% to the reference sequence.

### 2.5. Serotype, STs, and phylogenetic analysis

Serotype prediction was performed using the EnteroBase online analysis platform.^6^ The MLST 2.0 - Center for Genomic Epidemiology software^7^ was utilized to analyze the sequence types (STs) of all isolates through the application of multilocus sequence typing (MLST). The core genome multilocus sequence typing (cgMLST) analysis was performed using the 2513 locus cgMLST V1 + HierCC V1 scheme provided by EnteroBase. The construction of the cgMLST tree utilized the MSTree V2 algorithm from the cgMLST V1 + HierCC V1 scheme. To facilitate comparisons with the strains isolated in this study, the tested sequences derived from available ETEC genomes in the Enterobase database were included in the analysis for generating the cgMLST tree. A total of 102 ETEC genomes were carefully selected from 13 countries, including the United States, the United Kingdom, Canada, France, Germany and Denmark, etc. These bacteria genomes were sourced from diverse origins, including humans, wildlife, livestock, environment, poultry and food. The minimum spanning tree was visualized using the software GrapeTree (Zhou et al., 2018).

### 2.6. Data analysis

Heml 2.0 was employed to construct heatmaps displaying the distribution of ARGs, VFs, and plasmid incompatibility groups (Ning et al., 2022). The scoring scheme employed in this study assigned a value of 1 or 0 to denote the presence or absence of the designated gene, respectively.

## 3. Results

### 3.1. Molecular identification of ETEC by PCR

**Figure 1:**
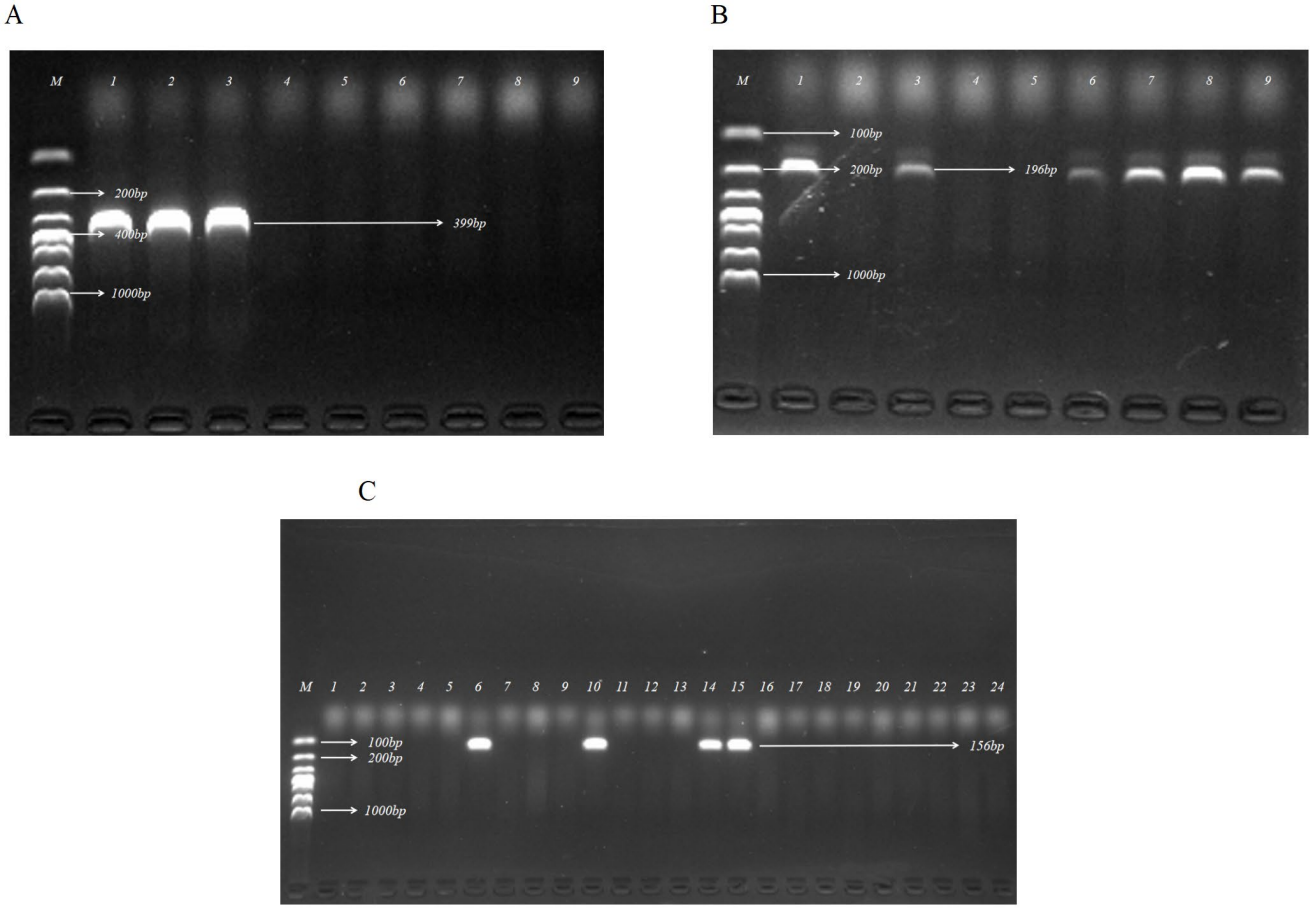
Identification of ETEC by PCR assay targeting enterotoxin genes of LT, STa, and STb. **(A)** Agarose gel electrophoresis of the PCR products for detection of LT in partial *E. coli* isolates. **(B)** Agarose gel electrophoresis of the PCR products for detection of STa in partial *E. coli* isolates. **(C)** Agarose gel electrophoresis of the PCR products for detection of STb in partial *E. coli* isolates. Lanes: A_1_, A_2_, A_3_, B_1_, B_3_, B_6_, B_7_, B_8_, B_9_, C_6_, C_10_, C_14_, C_15_, isolates that were positive for virulence factor genes; M, DL 1000 DNA Marker (100, 200, 300, 400, 500, 700, 1,000 bp).

To identify potential ETEC colonies, ETEC-specific LT and ST gene primers were applied in PCR detection. Figure 1 displays the agarose gel electrophoresis results for representative *E coli* isolates. In this study, a total of 19 ETEC isolates were confirmed out of 412 *E. coli* strains isolated from pig fecal samples, resulting in a recovery rate of 4.61%.

### 3.2. Antimicrobial resistance phenotypes

**Figure 2:**
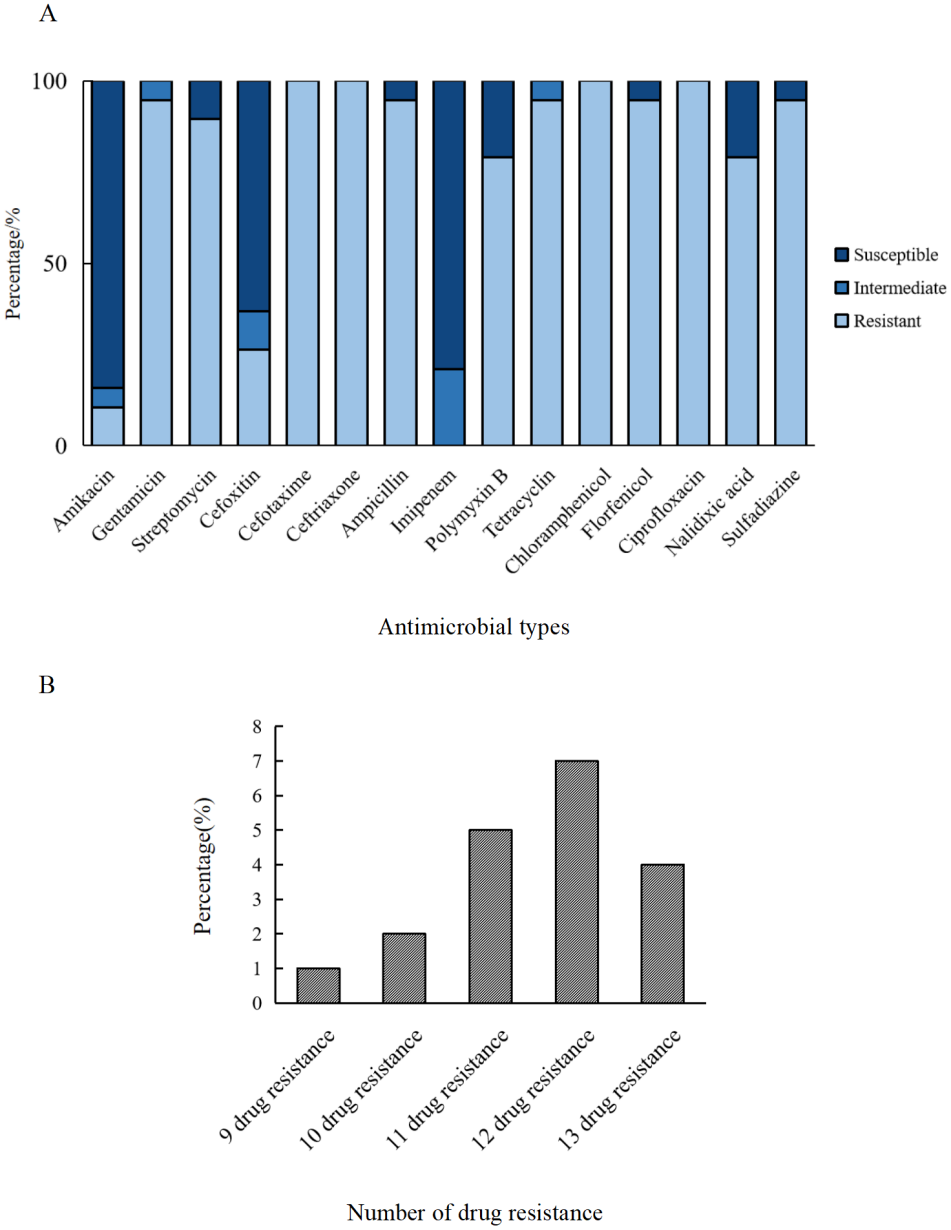
AMR characteristics of ETEC isolates from fecal samples of pigs with diarrhea in large-scale pig farms in Sichuan, China. **(A)** The ratio of susceptible, intermediate, and resistant strains of each antimicrobial agent. **(B)** The percentage of ETEC isolates with each type of MDR strain. Amikacin (AMK), gentamicin (GEN), streptomycin (STR), cefoxitin (FOX), cefotaxime (CTX), ceftriaxone (CRO), ampicillin (AMP), imipenem (IPM), polymyxin B (PMB), tetracycline (TET), chloramphenicol (CHL), florfenicol (FFC), ciprofloxacin (CIP), nalidixic acid (NAL), and sulfasalazine (SSZ).

**Figure 3:**
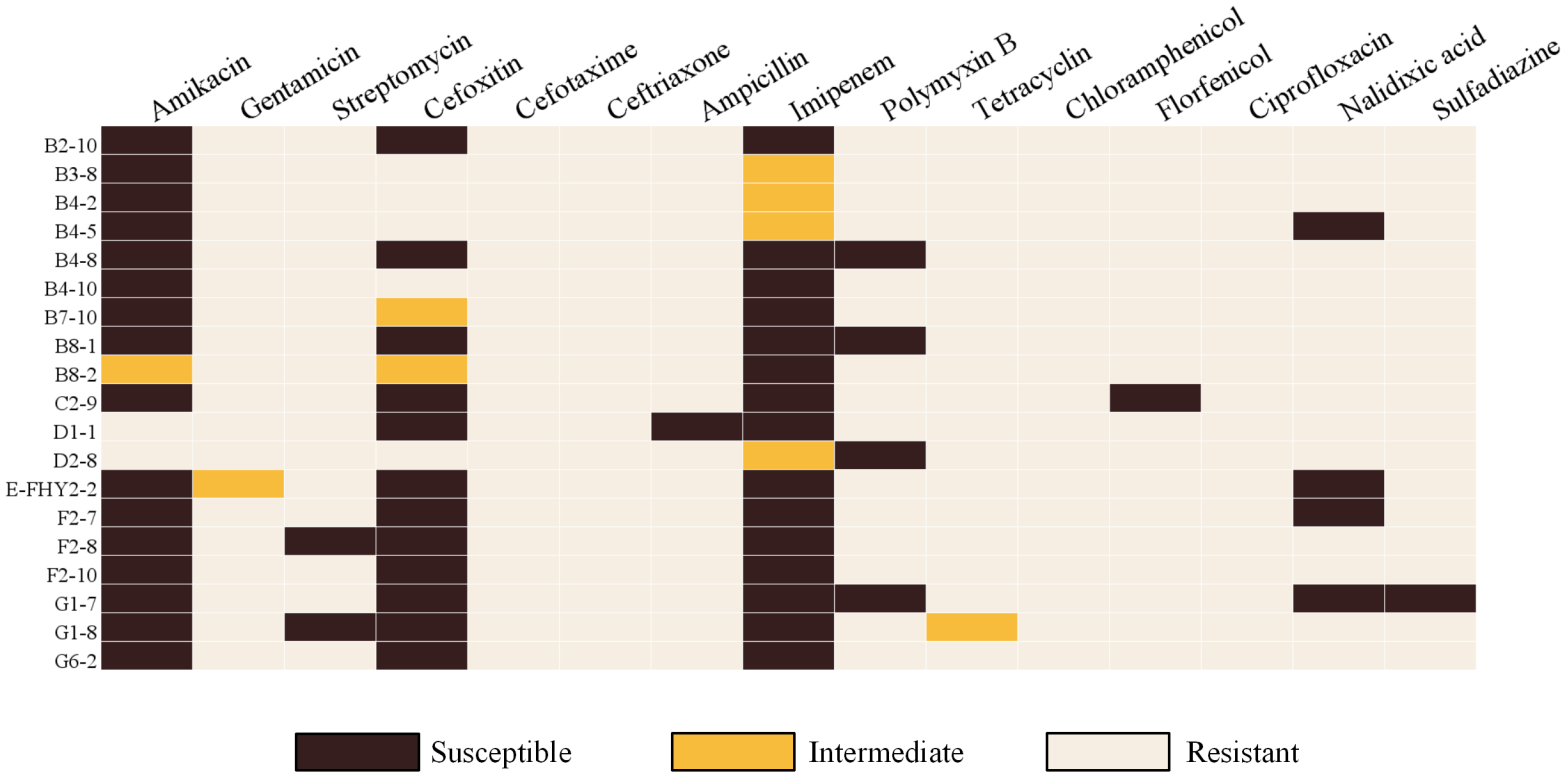
Heat map showing the results of antimicrobial susceptibility testing of ETEC isolates from fecal samples of pigs with diarrhea in large-scale pig farms in Sichuan, China. Dark brown indicates susceptible phenotype, and light brown indicates resistant phenotype.

The micro-broth dilution method was employed to assess the AMR of ETEC isolates, and the results demonstrated significant resistance to CTX (100%, 19/19), CRO (100%, 19/19), CHL (100%, 19/19), CIP (100%, 19/19), and over 94% resistance rates to GEN, AMP, TET, FFC, and SSZ (Figure 2A). In contrast, the isolates exhibited high susceptibility rates to AMK (84.21%, 16/19), IPM (78.95%, 15/19) and FOX (63.16%, 12/19). In addition, a marked divergence in AMR profiles among the isolates (Figure 3), with the predominant AMR profiles of GEN-STR-CTX-CRO-AMP-PMB-TET-CHL-FFC-CIP-NAL-SSZ (26.32%, 5/19), GEN-STR-FOX-CTX-CRO-AMP-PMB-TET-CHL-FFC-CIP-NAL-SSZ (15.79%, 3/19) and GEN-STR-CTX-CRO-AMP-TET-CHL-FFC-CIP-NAL-SSZ (10.53%, 2/19) (Table 2). Further evaluation of MDR isolates revealed that all strains were resistant to a minimum of 9 antimicrobial agents and a maximum of 13 antimicrobial agents, with the majority (36.84%, 7/19) of the isolates showed resistance to 12 antimicrobial agents, and a significant proportion (26.32%, 5/19) were found to be resistant to 11 antimicrobial agents (Figure 2B).

**Table 2:** AMR patterns of ETEC isolates.

Isolates	AMR patterns
B2-10	GEN-STR-CTX-CRO-AMP-PMB-TET-CHL-FFC-CIP-NAL-SSZ
B3-8	GEN-STR-FOX-CTX-CRO-AMP-PMB-TET-CHL-FFC-CIP-NAL-SSZ
B4-2	GEN-STR-FOX-CTX-CRO-AMP-PMB-TET-CHL-FFC-CIP-NAL-SSZ
B4-5	GEN-STR-FOX-CTX-CRO-AMP-PMB-TET-CHL-FFC-CIP-SSZ
B4-8	GEN-STR-CTX-CRO-AMP-TET-CHL-FFC-CIP-NAL-SSZ
B4-10	GEN-STR-FOX-CTX-CRO-AMP-PMB-TET-CHL-FFC-CIP-NAL-SSZ
B7-10	GEN-STR-CTX-CRO-AMP-PMB-TET-CHL-FFC-CIP-NAL-SSZ
B8-1	GEN-STR-CTX-CRO-AMP-TET-CHL-FFC-CIP-NAL-SSZ
B8-2	GEN-STR-CTX-CRO-AMP-PMB-TET-CHL-FFC-CIP-NAL-SSZ
C2-9	GEN-STR-CTX-CRO-AMP-PMB-TET-CHL-CIP-NAL-SSZ
D1-1	AMK-GEN-STR-CTX-CRO-PMB-TET-CHL-FFC-CIP-NAL-SSZ
D2-8	AMK-GEN-STR-FOX-CTX-CRO-AMP-TET-CHL-FFC-CIP-NAL-SSZ
E-FHY2-2	STR-CTX-CRO-AMP-PMB-TET-CHL-FFC-CIP-SSZ
F2-7	GEN-STR-CTX-CRO-AMP-PMB-TET-CHL-FFC-CIP-SSZ
F2-8	GEN-CTX-CRO-AMP-PMB-TET-CHL-FFC-CIP-NAL-SSZ
F2-10	GEN-STR-CTX-CRO-AMP-PMB-TET-CHL-FFC-CIP-NAL-SSZ
G1-7	GEN-STR-CTX-CRO-AMP-TET-CHL-FFC-CIP
G1-8	GEN-CTX-CRO-AMP-PMB-CHL-FFC-CIP-NAL-SSZ
G6-2	GEN-STR-CTX-CRO-AMP-PMB-TET-CHL-FFC-CIP-NAL-SSZ

AMK, amikacin; GEN, gentamicin; STR, streptomycin; FOX, cefoxitin; CTX, cefotaxime; CRO, ceftriaxone; AMP, ampicillin; IPM, imipenem; PMB, polymyxin B, TET, tetracycline, CHL, chloramphenicol; FFC, florfenicol, CIP, ciprofloxacin, NAL, nalidixic acid; SSZ, sulfasalazine.

### 3.3. Presence of ARGs and chromosomal point mutations in ETEC

**Figure 4:**
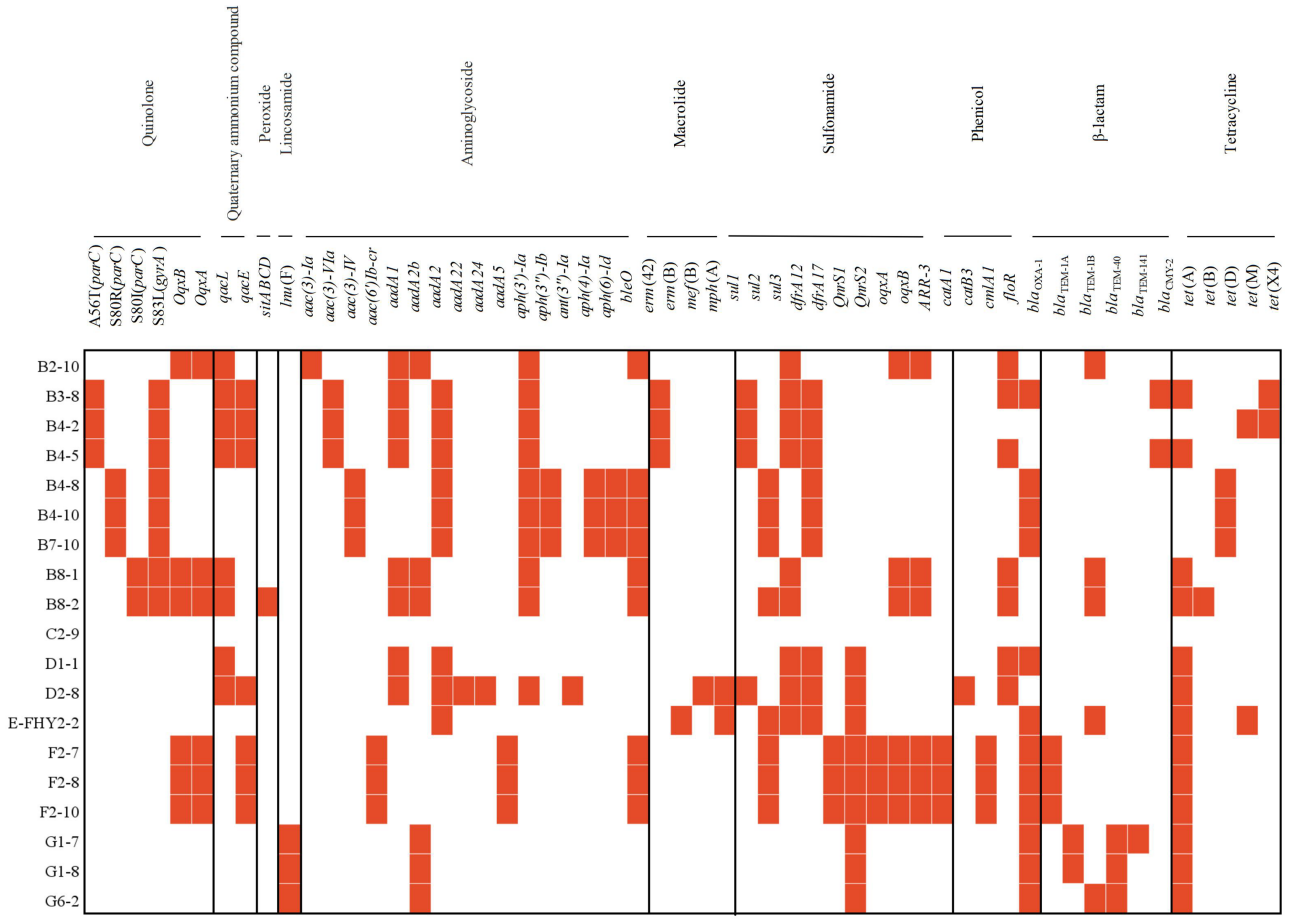
Heat map showing the acquired ARGs or chromosomal point mutation profiles of ETEC isolates from fecal samples of pigs with diarrhea in large-scale pig farms in Sichuan, China.

Totally, 53 ARGS were detected in ETEC isolates. The most frequently detected resistance gene was *tet*(A) (68.42%, 13/19), which encodes a tetracycline transporter protein that reduces the concentration of tetracycline by transporting it from the inside to the outside of the cell. Additionally, other ARGs, including *floR* (63.16%, 12/19), *aph(3′)-Ia* (52.63%, 10/19), *aadA2* (47.37%, 9/19), *bleO* (47.37%, 9/19), *sul3* (47.37%, 9/19), *dfrA12* (47.37%, 9/19), and *QnrS1* (47.37%, 9/19), provided resistance to several antimicrobial agents, such as quinolones, aminoglycosides, chloramphenicol and sulfonamides. It is important to note that these ARGs may exhibit cross-resistance to other antibiotic classes, consequently reducing the effectiveness of other drugs. Alarmingly, the presence of *tet*(X4) was detected in 2 isolates (10.53%), indicating the presence of the gene associated with high-level tigecycline resistance. This finding highlighted the emergence of plasmid-mediated tigecycline resistance genes (Sun et al., 2019). The quinolone resistance determining region (QRDR) is a specific segment of the bacterial genome that contains certain regions of the *gyrA* and *parC* genes, which are critical sites for the interaction of quinolones with bacterial DNA topoisomerases. Mutations in the QRDR could lead to the development of resistance to quinolones. Chromosomal point mutations in QRDR were observed in both *gyrA* (42.11%, 8/19) and *parC* (42.11%, 8/19) genes in ETEC isolates. The exclusively observed mutation in *gyrA* was S83L (100%), whereas mutations in *parC* were commonly detected as S80R (37.5%), A56T (37.5%), and S80I (25%). Importantly, the genotypes of the isolates were fundamentally aligned with their antimicrobial resistance phenotypes (Figure 4).

### 3.4. Virulence-associated determinants analysis

**Figure 5:**
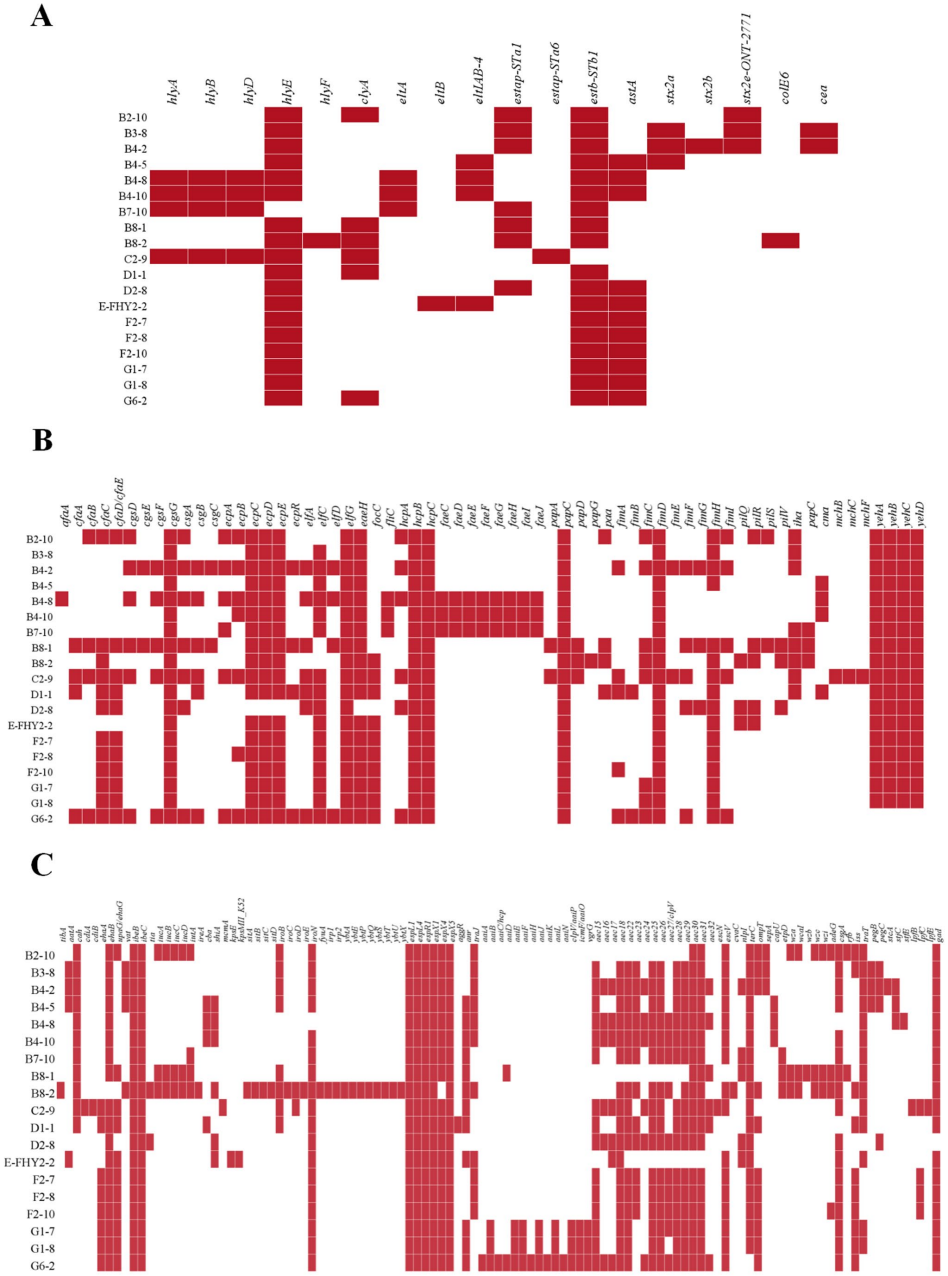
Heat map showing the virulence gene in ETEC isolates from fecal samples of pigs with diarrhea in large-scale pig farms in Sichuan, China. **(A)** The virulence genes associated with toxins. **(B)** The virulence genes associated with adherence. **(C)** The remaining virulence genes not associated with toxins or adherence.

The genetic analysis of virulence determinants in VFDB has revealed the presence of a diverse set of virulence genes in 19 isolates of ETEC that fall into 13 different types, totaling 195 genes (Figure 5), whose functions included adherence, autotransporter, invasion, iron uptake, non-LEE encoded TTSS effectors, regulation, secretion system, toxin, antiphagocytosis, biofilm formation, serum resistance, fimbrial adherence determinants and glutamate decarboxylase. Adherence genes constituted the largest category, with a total of 65 virulence genes identified in ETEC (Figure 5B). Adherence genes played a vital role in ETEC pathogenesis, encoding various adhesins that facilitate bacterial attachment to host cells and the establishment of infection. The diverse adherence genes, such as afimbrial adhesin AFA-I, CFA/I fimbriae, curli fibers, *E. coli* common pilus (ECP), *E. coli* laminin-binding fimbriae (ELF), *eaeH*, F1C fimbriae, hemorrhagic *E. coli* pilus (HCP), K88 fimbriae, P fimbriae, porcine attaching-effacing associated protein, type I fimbriae, and type IV pili, suggested that ETEC has evolved multiple mechanisms to adhere to host cells and evade host immune defenses (Table 3).

**Table 3:** Virulence gene in ETEC isolates.

VF class	Virulence factors	Related genes
Adherence	Afimbrial adhesin AFA-I	*afaA*
	CFA/I fimbriae	*cfaA/B/C/D/E*
	Curli fibers	*cgsA/B/C/D/E/F/G*
	*E. coli* common pilus (ECP)	*ecpA/B/C/D/E/R*
	*E. coli* laminin-binding fimbriae (ELF)	*elfA/C/D/G*
	EaeH	*eaeH*
	F1C fimbriae	*focC*
	Flagella (cluster I) (*Yersinia*)	*fliC*
	Hemorrhagic *E. coli* pilus (HCP)	*hcpA/B/C*
	K88 fimbriae	*faeC/D/E/F/G/H/I/J*
	P fimbriae	*papA/C/D/G*
	Porcine attaching-effacing associated protein	*paa*
	Type I fimbriae	*fimA/B/C/D/E/F/G/H/I*
	Type IV pili (*Yersinia*)	*pilQ/R/S/V*
	Adherence protein	*iha*
	Outer membrane usher P fimbriae	*papC*
	Colicin M activity	*cma*
	MchC protein	*mchB/C/F*
	YHD fimbriael cluster	*yehA/B/C/D*
Autotransporter	AIDA-I type	*tibA*
	AatA	*aatA*
	Cah	*cah*
	Contact-dependent inhibition CDI system	*cdiA/B*
	EhaA/B	*ehaA/B*
	UpaG adhesin	*upaG/ehaG*
	Vacuolating autotransporter gene	*vat*
Invasion	Invasion of brain endothelial cells (Ibes)	*ibeB/C*
	Tia/Hek	*tia*
Iron uptake	Aerobactin siderophore	*iucA/B/C/D, iutA*
	Iron-regulated element	*ireA*
	Colicin B	*cba*
	Homologs of the *Shigella flexneri* SHI-2 pathogenicity island gene *shiA*	*shiA*
	Microcin M part of colicin H	*mcmA*
	Polysialic acid transport protein	*kpsE, kpsMII_K52*
	Iron/manganese transport	*sitA/B/C/D*
	Salmochelin siderophore	*IroB/C/D/E/N*
	Yersiniabactin siderophore	*fyuA, irp1/2, ybtA/E/P/Q/S/T/U/X*
Non-LEE encoded TTSS effectors	EspL1/L4/R1/X1/X4/X5	*espL1/L4/R1/X1/X4/X5*
Regulation	AggR	*aggR*
	AraC negative regulator	*anr*
	Protein TraJ (Positive regulator of conjugal transfer operon)	*traJ*
Secretion system	AAI/SCI-II T6SS	*aaiA/B/C/D/E/F/H/J/K/L/N/P/O,hcp,clpV,icmF,vgrG*
	AEC T6SS	*aec15 ~ 18, aec22 ~ 32*
	LEE locus encoded TTSS	*escN/V*
	Microcin C	*cvaC*
	Lipoprotein NlpI precursor	*nlpI*
	Tellurium ion resistance protein	*terC*
	Outer membrane protease (protein protease 7)	*ompT*
	Serine protease autotransporters of *Enterobacteriaceae* (SPATE)-*Shigella* extracellular protein A	*sepA*
	Hexosyltransferase homolog	*capU*
	Type II secretion protein *EtpD*	*etpD*
Toxin	Alpha-hemolysin	*hlyA/B/D/E/F*
	Hemolysin/cytolysin A	*clyA*
	Heat-labile enterotoxin	*eltA, eltB, eltIAB-4*
	Heat-stable enterotoxin	*estap-STa1, estap-STa6, estb-STb1, astA*
	Shiga-like toxin	*stx2a, stx2b, stx2e-ONT-2771*
	Colicin E6	*colE6*
	Colicin E1	*cea*
Antiphagocytosis	Capsule (*Klebsiella*)	*wza/b/c/i,wcaI*
Biofilm formation	AdeFGH efflux pump/transport autoinducer (*Acinetobacter*)	*adeG*
	Curlin major subunit CsgA	*csgA*
Serum resistance	LPS rfb locus (*Klebsiella*)	*rfb*
	Increased serum survival	*iss*
	Outer membrane protein complement resistance	*traT*
Fimbrial adherence determinants	Peg (*Salmonella*)	*pegB/C*
	Stc (*Salmonella*)	*stcA*
	Stj (*Salmonella*)	*stjC*
	Stf (*Salmonella*)	*stfE*
	Lpf (*Salmonella*)	*lpfB/C/E*
Glutamate decarboxylase	GAD	*gad*

The investigation revealed the presence of a diverse range of LT genes, namely *eltA*, *eltB*, and *eltIAB-4*, as well as ST genes, encompassing *estap-STa1*, *estap-STa6*, *estb-STb1*, and *astA*, within the identified ETEC isolates (Figure 5A). Notably, the detection rate of *estb-STb1* exhibited the highest frequency, observed in 18 isolates (94.74%), while *astA* was found in 11 isolates (57.89%). In contrast, the presence of *eltB* and *estap-STa6* was exceptionally low, with only one isolate (5.26%) exhibiting their presence. STEC is distinguished by the presence of *stx1* or *stx2* genes. The isolates analyzed in this study exhibited the detection of *stx2* genes, including *stx2a* (15.79%), *stx2b* (5.26%), and *stx2e-ONT-2771* (15.79%). The 4 isolates (21.05%), which carried *stx* genes, were identified as hybrid strains.

Furthermore, it is noteworthy that the genes of *cgsG*, *elfG*, *hcpB*, *hcpC*, *papC*, *fimD*, *ibeB*, *ibeC*, *espL1*, *espL4*, *espR1*, *espX1*, and *espX5* were present in all 19 ETEC isolates, indicating their conserved and essential role in the pathogenesis and virulence of ETEC. The identified genes encoded a variety of proteins, including membrane proteins, chaperones, invasion-related proteins, effector proteins of type III secretion system, transcriptional regulators and proteins essential for the assembly and secretion of fimbriae. These gene products function in membrane transport, protein folding, invasion and fimbriae biogenesis, among other cellular processes.

### 3.5. Plasmid incompatibility groups

**Figure 6:**
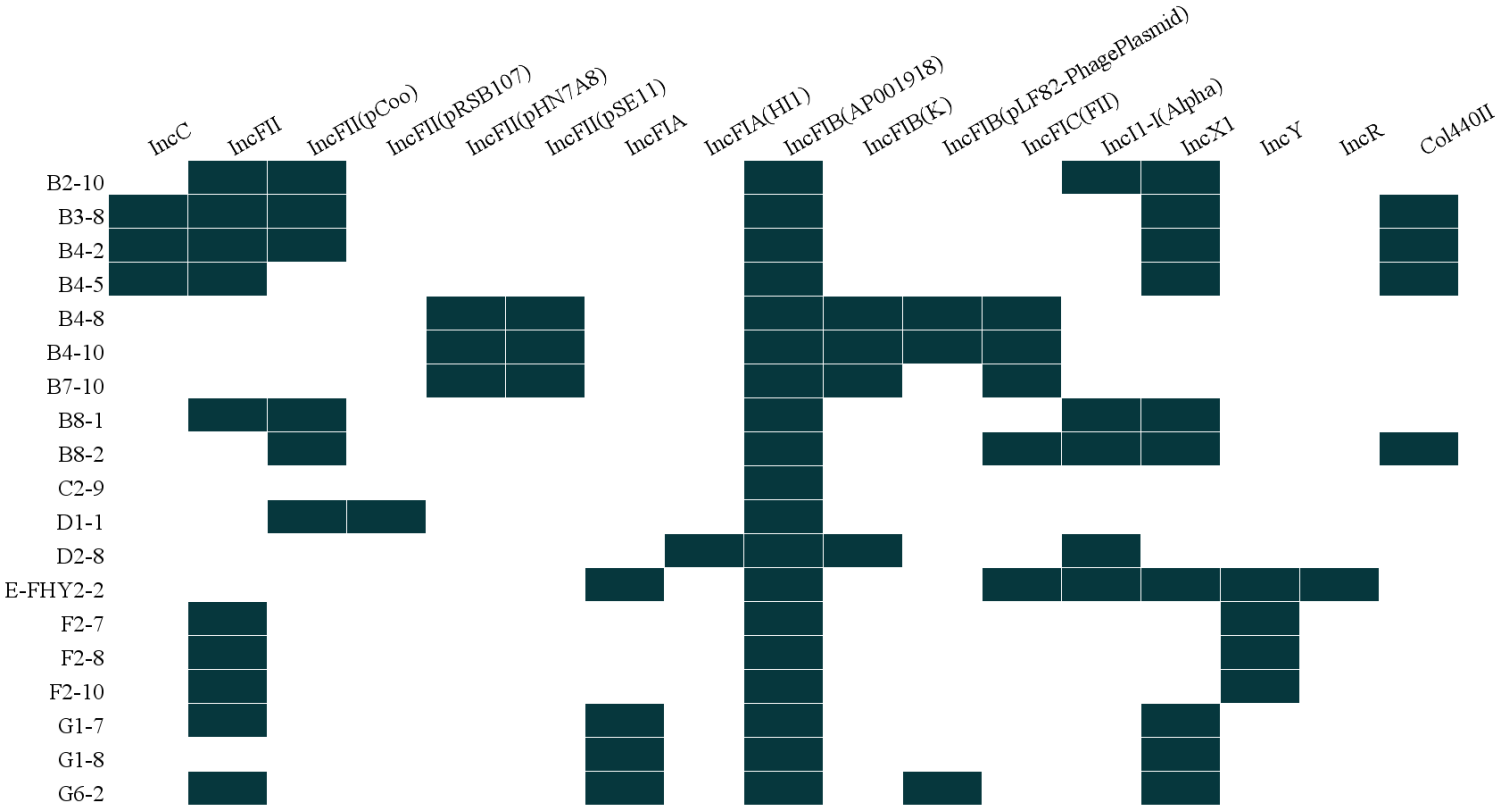
Heat map showing the plasmid replicon profiles for ETEC isolates from fecal samples of pigs with diarrhea in large-scale pig farms in Sichuan, China.

The results indicated that a diverse array of plasmid replicons was identified among ETEC isolates, with 17 distinct types being detected. Notably, all 19 isolates were found to harbor at least one plasmid replicon-associated gene, with multiple replicons being detected in 94.74% (18/19) of the isolates. The distribution of strains within the incompatibility group exhibited variability, with one strain containing a single replicon (5.26%), while others harbored 3, 4, 5, 6, or 7 replicons, accounting for 26.32%, 10.53%, 26.32%, 26.32%, and 5.26% of the isolates, respectively. These observations suggested that the majority of ETEC isolates contained at least two or more plasmids, with IncFIB (AP001918) being a plasmid replicon type present in all bacterial strains (100%, 19/19), followed by IncFII and IncX1 plasmids, both of which were detected in 52.63% (10/19) of the isolates (Figure 6).

### 3.6. Serotype, STs, and phylogenetic analysis

Through Illumina sequencing, 19 MDR ETEC strains were examined, revealing a diverse group of O-serogroups and H-serogroups, that serotypes O22:H10 (10.53%, 2/19), O163orOX21:H4 (10.53%, 2/19), and O105:H8 (10.53%, 2/19) were dominant. The isolates underwent MLST analysis to determine the STs. The analysis was successful for 18 isolates, revealing the presence of 9 different STs. However, one isolate could not be defined. Among the identified STs, ST13 (15.79%, 3/19), ST100 (15.79%, 3/19), ST206 (15.79%, 3/19), and ST336 (15.79%, 3/19) were found to be the most common (Table 4).

**Table 4:** Serotype and sequence types of multidrug resistant ETEC isolates.

Strains	O antigen	H antigen	ST type
B2-10	O9	H4	953
B3-8	O9	H29	206
B4-2	O88	H29	206
B4-5	O8	H29	206
B4-8	O45	H10	100
B4-10	O22	H10	100
B7-10	O22	H10	100
B8-1	O163orOX21	H4	953
B8-2	O163orOX21	H4	–
C2-9	O149	H7	1,403
D1-1	O141	H21	683
D2-8	O141	H26	1,112
E-FHY2-2	O141	H38	5,420
F2-7	O13	H16	336
F2-8	O128	H16	336
F2-10	O105	H16	336
G1-7	O105	H8	13
G1-8	O105	H8	13
G6-2	–	H8	13

**Figure 7:**
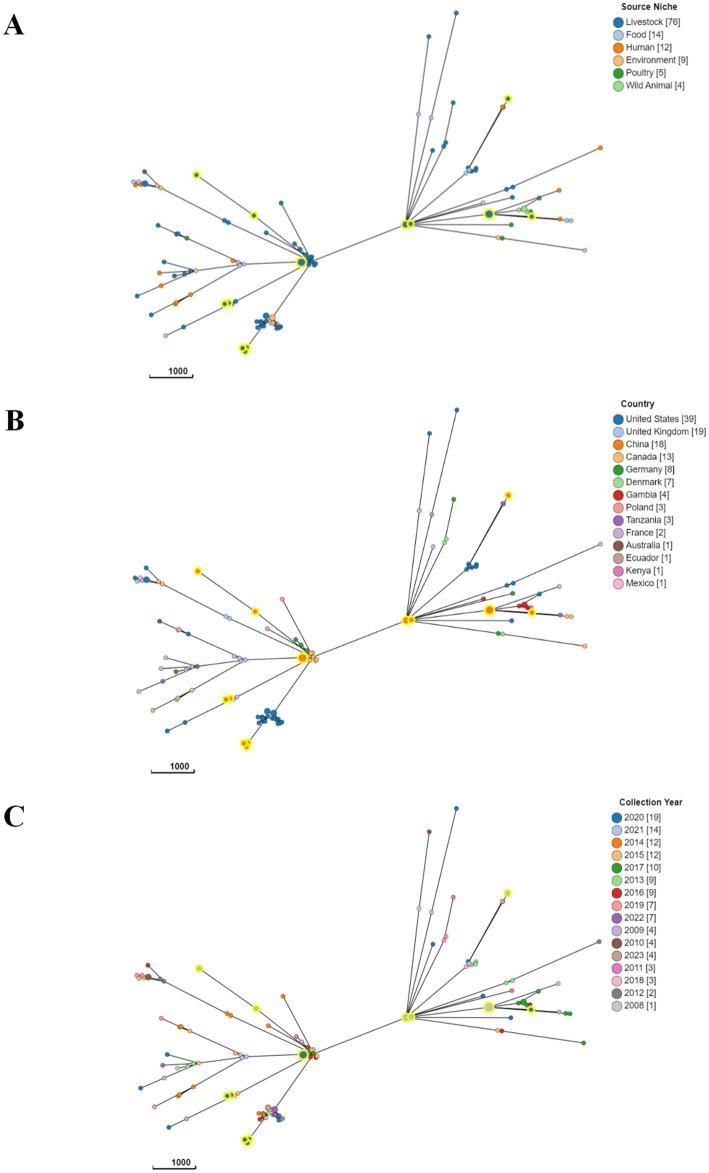
A core genome minimum spanning tree was generated to demonstrate the phylogenetic relationship of ETEC based on sources **(A)**, country of origin **(B)**, and time of collection **(C)**. A minimum spanning tree for 121 ETEC isolates was generated from cgMLST data the 2513 locus cgMLST V1 + HierCC V1 scheme, which is available within EnteroBase. The isolates were annotated by their **(A)** sources, **(B)** country, **(C)** year of isolation. MSTree V2 tree was created based on cgMLST, with nodes colored according to clusters defined in the study. The isolates characterized in the study were circled with a large yellow halo for ease of identification.

In order to investigate the phylogenetic characteristics of ETEC isolates, a minimum spanning tree was constructed using cgMLST analysis to classify 121 ETEC isolates into 2 clusters (Figure 7). However, the minimum spanning tree did not reveal any obvious relationships between the observed STs. Remarkably, diverse sources had isolates from the same cluster, indicating the widespread dissemination of ETEC among animals, food, and humans (Figure 7A). The results suggested that the lineage of ETEC isolates was not significantly associated with geographical distribution, as different types of STs were widely distributed in various countries (Figure 7B). Moreover, no significant pattern was observed in the prevalence of STs across different years (Figure 7C).

## 4. Discussion

Diarrhea resulting from ETEC infection is prevalent among pigs and imposes significant economic burdens to the pig industry globally. The ensuing diarrhea in piglets is largely attributed to ETEC and can have detrimental effects on growth and production performance, presenting a significant technological hurdle for contemporary swine production (Gresse et al., 2017). However, despite its importance, the genomic characteristics and variation of porcine-origin ETEC have been poorly investigated. To address this gap, WGS was applied to identify ETEC isolates from pig feces samples obtained from large-scale farms in Sichuan, China. Our study aimed to clarify the serotypes, AMR, ARGs, VFs, and plasmid incompatibility types of ETEC associated with diarrhea in pigs.

ETEC strains have significant implications for both animal and human health. In pigs, ETEC colonization can lead to severe enteric diseases, resulting in adverse impacts on growth rates, feed conversion, and economic losses in the livestock industry. Similarly, in humans, ETEC is known to cause acute diarrhea, particularly affecting vulnerable populations like infants, young children and individuals in developing regions with limited access to clean water and sanitation facilities. Notably, ETEC is a prominent causative agent of traveler’s diarrhea, posing a substantial threat to human health (Guiral et al., 2019). Effective measures to mitigate ETEC transmission encompass improved sanitation practices, access to clean water, and adherence to proper hygiene protocols for both animal and human populations. Furthermore, implementing targeted vaccination programs that specifically address ETEC strains can significantly contribute to infection prevention and overall reduction in disease burden. Hence, it is of paramount importance to gain insights into the AMR profiles and complete genomic characteristics of ETEC.

In the present study, the predicted dominant serotypes in large-scale pig farms in Sichuan Province of China were O105, O141, H8, H10, H16 and H29, which differed from previous studies conducted in other regions of China. For example, in large-scale pig farms in northern Jiangsu, the dominant serotypes of ETEC were O8, O101, and O128 (Yang et al., 2020). Similarly, in certain areas of Anhui Province, pig farms showed a prevalence of O8, O20, and O128 as the predominant serotypes of ETEC, with generally high levels of virulence (Ren et al., 2020). These results indicated that ETEC with a diverse group of serotypes were circulating in pigs, with significant diversity in serotypes observed both within and across regions. Furthermore, our WGS analysis revealed the distribution of ST100 among MDR ETEC isolates from pig farms, which was considered to be a classic strain of porcine ETEC (non-zoonotic animal pathogen) (Abraham et al., 2015). Existing studies have demonstrated that the O149 serogroup (ST100) is the predominant cause of PWD in Australian pigs (Abraham et al., 2014). The variety of serotypes in ETEC is significant for disease severity, surveillance and vaccine development. It helps track strain spread, identify outbreaks, and select relevant antigens for vaccines targeting specific serotypes.

The analysis encompassed a comprehensive evaluation of 121 ETEC isolates worldwide utilizing cgMLST analysis, a highly precise method for subtype characterization based on genomic variations. The minimum spanning tree, derived from this analysis, provided insights into the genetic relatedness among ETEC isolates. However, the analysis failed to reveal distinct patterns of relatedness, as different STs were dispersed across various clusters. Furthermore, the same cluster accommodated isolates originating from diverse sources, including animals, environment, food and humans. These results indicated a wide distribution of ETEC across multiple reservoirs and transmission routes, with limited association between the genetic diversity of ETEC isolates and their geographical or temporal origins.

The absence of conspicuous patterns in the cgMLST data may be attributed to a variety of underlying factors, collectively contributing to the elusive nature of the observed relationships. Primarily, the substantial genetic diversity prevalent among ETEC isolates unquestionably played a pivotal role in the intricate network of associations within their genomic profiles. ETEC displays notable genetic diversity that emerges from mechanisms like mutations and genetic rearrangements. Consequently, distinct sets of genes, including those encoding VFs or AMR, can be found among ETEC strains. The extensive range of genetic variations posed significant challenges in deciphering the definitive relationships among these isolates. Moreover, the recurrent occurrence of horizontal transfer events involving mobile genetic elements further complicates the genomic makeup of ETEC isolates. These mobile genetic elements have the capacity to move across bacterial strains, leading to a mosaic-like configuration of genomes. This facilitates the exchange of genetic material, including VFs and ARGs, thereby shaping the genetic composition of ETEC populations. These genetic exchange events tended to blur the demarcations between different lineages, thereby obscuring any anticipated straightforward associations. Furthermore, the complex evolutionary history of this pathogenic microorganism, sculpted by diverse selective pressures, potentially exerts a profound impact on the observed genetic patterns. Adaptation plays a key role, driving the development of traits that enhance survival and reproduction in specific environments. In the case of ETEC, adaptation enables colonization of host environments, evasion of immune responses, and acquisition of nutrients. Genetic drift, a stochastic process, introduces random fluctuations in allele frequencies over time, leading to the divergence of lineages and contributing to genetic diversity. The combined effects of adaptation, genetic drift and other forces ultimately contribute to the observed genetic patterns, influencing the evolution and diversity of ETEC populations. Consequently, the resulting intricate genetic architecture posed a significant obstacle in identifying unambiguous associations within the cgMLST data.

*In vitro* antimicrobial susceptibility testing is a crucial tool providing guidance for veterinary clinical practice. The detection of MDR ETEC isolates from pigs with diarrhea on farms raised a concerning situation of AMR. The study demonstrated that all ETEC isolates were MDR. In comparison, the resistance rates of ETEC strains collected in Bangladesh to ampicillin (66%), cotrimoxazole (46%), doxycycline (44%), erythromycin (96%), nalidixic acid (83%), streptomycin (48%) and tetracycline (42%) differed to some extent from our findings (Begum et al., 2016). Geographic variation in antimicrobial usage and resistance patterns is a likely explanation, as different regions may have different levels of antibiotic use in agriculture, leading to variations in the prevalence and types of resistant bacteria. Local antibiotic prescription practices and veterinary antibiotic use also contribute to these differences. Additionally, factors such as the specific strains of ETEC circulating in different populations, the genetic mechanisms of resistance acquired by the bacteria, and variations in surveillance methods used in different investigations can contribute to the disparities in resistance rates observed. Despite the ban on the usage of CHL in animal production in China since 2002 (Peng et al., 2022), CHL-resistant isolates were found on pig farms in Sichuan in this study, potentially attributed to continued use of FFC, a member of the chloramphenicol class (Yang et al., 2019). In contrast, IPM-resistant isolates were not found in the study, most likely due to the official policy prohibiting the use of carbapenem in livestock in China. Although the ban on AMK usage in agriculture in 2017 resulted in a low percentage (10.53%, 2/19) of AMK-resistant isolates on pig farms, the high PMB resistance rate (78.95%, 15/19) of ETEC isolates in this study indicated that drug misuse in pig industry is still of a significant concern (Shen et al., 2020; Wang et al., 2020).

Fortunately, despite the majority of strains showing resistance to PMB, *mcr-1* gene was not observed in the present study, which was first identified in 2016 in bacterial isolates from animal foods (chickens and pigs) in China (Liu et al., 2016). This plasmid-mediated resistance gene encodes an enzyme that belongs to the phosphoethanolamine transferase family and is responsible for synthesizing and conjugating pEtN to lipid A (Ayoub Moubareck, 2020). Other *mcr* genes, including *mcr-2* and *mcr-3*, have also been found in plasmids among enterobacteria (Zhang et al., 2018). These resistance genes suggested the presence of various pathways for the horizontal transmission of colistin resistance, which could lead to its high potential for dissemination. Based on our results, it was suspected that the PMB-resistance observed in the ETEC isolates may not be attributed to horizontal transfer. Longitudinal studies in China found that reduced colistin sales were associated with decreased occurrence of *mcr-1*-producing *E. coli* in pig feces and human intestines. Human infections with colistin-resistant *E. coli* also declined after 2017, possibly due to the Chinese government’s ban on colistin as an animal growth promoter (Ewers et al., 2022).

It is worth noting that we detected the presence of *tet*(X4) in 2 (10.53%) isolates of ETEC. According to previous research, *E. coli* strains carrying *tet*(X4) may pose a risk of transmission to humans, as they exhibited a significant genetic resemblance to human commensal *E. coli*. Although *tet*(X4) was not extensively distributed across the country, it was highly endemic in northwestern China (Sun et al., 2020). Among 19 ETEC isolates, *tet*(A) was the most frequently detected ARGs, accounting for 68.42%. One potential explanation for the relatively low prevalence of tigecycline-resistance gene was the presence of TET-related ARGs, such as *tet*(A), *tet*(B), *tet*(C), and/or *tet*(M), which are often carried by *E. coli* strains from animals (Foong et al., 2020). The global spread of colistin-resistant *E. coli* and CRE poses major public health challenges. Consequently, tigecycline has become a crucial last-resort antibiotic for treating certain bacterial infections (Zhang et al., 2022). Given the emergence of tigecycline resistance mediated by *tet*(X4), our findings underscored the urgent need for heightened surveillance and intervention measures to prevent a potential dissemination of this ARGs.

Identifying the VFs of ETEC in pigs is crucial for understanding its pathogenicity and transmission, as well as developing effective prevention and treatment strategies. This study identified several essential genes, including c*gsG*, *elfG*, *hcpB*, *hcpC*, *papC*, *fimD*, *ibeB*, *ibeC*, *espL1*, *espL4*, *espR1*, *espX1*, and *espX5*, that play a significant role in pathogenesis and virulence. The presence of these genes in all ETEC isolates demonstrated their critical role in ETEC virulence. Furthermore, the investigation unveiled the capacity of the ETEC isolates to potentially produce either ST alone or both LT and ST toxins concurrently. Additionally, this observation underscored the significance of multiple virulence genes, such as *eltA*, *eltB*, *eltIAB-4*, *estap-STa1*, *estap-STa6*, *estb-STb1*, and *astA*, in potential toxin synthesis. STa has been implicated in human disease, while STb is predominantly associated with ETEC infection in pigs, although the presence of the *stb* gene has been observed in human ETEC isolates. Previous studies have shown that STa4 is frequently linked to diarrhea (Leonard et al., 2016). Plasmids are the predominant site for the presence of STa (von Mentzer et al., 2014), and the transfer of plasmids carrying ST toxin genes *sta* between different strains of *E. coli* is possible (Turner et al., 2006). Two distinct genetic variants of STa, namely STp and STh, are identified in human ETEC isolates. The findings indicate that STa3/4 (STh)-producing strains are correlated with disease in children, while STa5 (STp) strains are associated with disease in adults (Joffré et al., 2016). Extensive investigations have established the presence of *astA* gene, which encodes the enteroaggregative heat-stable enterotoxin EAST1 in ETEC linked to the occurrence of diarrheal disease in swine (Renzhammer et al., 2020). However, the precise role of this toxin in the disease remains a subject of ongoing debate, as neither EAST1-positive strains nor culture supernatants consistently induce explicit diarrhea in animal models (Dubreuil, 2019). Notably, a significant proportion of the strains examined in this study, specifically 57.89% (11/19) of the analyzed isolates, exhibited the presence of *astA* gene. This finding highlighted the widespread occurrence of this critical genetic determinant among ETEC isolates. Adhesin-related VFs, such as ECP, ELF, and HCP, also contributed to ETEC pathogenicity by interacting with enterotoxin, while Shiga-like toxin-related virulence factors, including *stx2a*, *stx2b*, and *stx2e-ONT-2771*, were identified with enterotoxic, cytotoxic and neurotoxic effects (Ahsan et al., 2020). In addition, the emergence of four hybrid isolates were confirmed in this study, which may be attributed to the frequent incidence of horizontal gene transfer (HGT) events. During HGT, specific genetic backgrounds acquire pathotype-defining virulence genes that are frequently harbored on mobile genetic elements (Leonard et al., 2016). *Stx2e*, in particular, is a crucial toxin responsible for edema disease in piglets, which often accompanies piglet diarrhea (Tseng et al., 2014). Hence, analyzing piglet symptoms alone is inadequate for diagnosing the disease accurately. A comprehensive investigation of ETEC VFs can provide an in-depth examination for the development of effective diagnostic methods and multivalent vaccines with extensive protection against this pathogen and other similar bacterial infections.

The acquisition of ARGs is facilitated by a diverse array of mobile genetic elements. Among these elements, plasmids are particularly significant in promoting ARGs dissemination (Bennett, 2008). The assessment of plasmid diversity in diverse cohorts can be achieved by utilizing plasmid incompatibility groups. The present study revealed significant differences in incompatibility groups prevalent in large-scale pig farms in southwestern China. The dissemination of epidemic plasmids has been linked to specific genes, and in this study, common replicators such as IncFIB, IncFII, and IncX1 were identified. Furthermore, certain groups of these plasmids, such as IncC, IncFIB, IncX1, IncFIIA, and IncY, have the ability to carry transfer, multidrug resistance and virulence functions across a broad range of hosts (Johnson and Nolan, 2009). IncFII has been recognized as a plasmid that disseminates multiple drug resistance (Wagner et al., 2014), and IncF is implicated in the transmission of drug resistance and virulence genes of *Enterobacteriaceae* (Hopkins et al., 2006). The possibility of transmission of MDR *E. coli* strains, especially those that produce extended-spectrum β-lactamases (ESBLs), from animals to humans is a growing concern (Jackson et al., 2015). IncC and IncX plasmid types have been recently reported as the most common among carbapenem-resistant *E. coli* (Kopotsa et al., 2019). This study found that the presence of the IncC plasmid was exclusive to hybrid strains, which was in line with the existence of *tet*(X4) gene. It was previously reported that the existence of a 119-kb multi-virulence IncFII/IncX1 hybrid ETEC/STEC plasmid (p15ODTXV) that carried virulence genes of both ETEC and STEC pathotypes played a pivotal role in the emergence of hybrid pathotypes, emphasizing the significance of plasmids in this mechanism (Brilhante et al., 2019). In this study, a comparable multi-virulence IncFII/IncX1 hybrid ETEC/STEC plasmid in the hybrid strain was clarified, providing additional evidence for the significant role of plasmids in the emergence of hybrid pathotypes. The transfer and transmission of plasmids played a critical role in the dissemination of virulence and drug resistance genes in pathogenic *E coli.* Plasmid transmission from animals to humans can lead to the spread of AMR, zoonotic infections, increased virulence and challenges in treating ETEC infections. Thus, understanding the incompatibility groups of porcine ETEC plasmids can inform the development of strategies to control and prevent ETEC infections in pigs.

## 5. Conclusion

In this study, an investigation was employed to analyze ETEC isolates from pigs with diarrhea in China. ETEC isolates exhibited notable levels of multidrug resistance, with the emergence of mobile *tet*(X4) genes in specific hybrid isolates, posed a challenge to swine industry and public health. Moreover, the study identified multiple plasmids capable of facilitating the horizontal transfer of multidrug resistance and virulence genes across diverse host organisms, which has the potential to exacerbate the dissemination of resistance traits, thereby posing a substantial threat to the swine industry. In addition, the strains under investigation were found to harbor various VFs associated with the pathogenesis and severity of animal diseases. The absence of conspicuous patterns in the cgMLST data among the ETEC isolates, suggested their wide distribution across different reservoirs and transmission routes. These results demonstrated the urgent need for intensified surveillance measures to effectively monitor the emergence, resistance and potential transmission of ETEC among porcine herds in China.

## Data availability statement

The datasets generated for this study can be found in the NCBI Bioproject with the accession number PRJNA983238.

## Author contributions

JH: investigation, software, data curation, writing-original draft, and funding acquisition. JL: formal analysis. XHu and QZ: methodology. JX, MC, YX, and SC: writing-review and editing. YH and YW: visualization. LZ: methodology, supervision, review and editing, and funding acquisition. XHa: conceptualization, project administration, resources, and funding acquisition. All authors contributed to the article and approved the submitted version.

## Funding

This work was supported by the grant from Natural Science Foundation of Sichuan, China (2023NSFSC0175) and Sichuan Science and Technology Program (2022ZDZX0017).

## Conflict of interest

The authors declare that they have no known competing financial interests or personal relationships that could have appeared to influence the work reported in this paper.

## Publisher’s note

All claims expressed in this article are solely those of the authors and do not necessarily represent those of their affiliated organizations, or those of the publisher, the editors and the reviewers. Any product that may be evaluated in this article, or claim that may be made by its manufacturer, is not guaranteed or endorsed by the publisher.

## Footnotes

^1^ https://card.mcmaster.ca/

^2^ https://cge.food.dtu.dk/services/ResFinder/

^3^ http://www.mgc.ac.cn/cgi-bin/VFs/v5/main.cgi

^4^ https://cge.food.dtu.dk/services/VirulenceFinder/

^5^ https://cge.food.dtu.dk/services/PlasmidFinder/

^6^ https://enterobase.warwick.ac.uk/

^7^ https://cge.food.dtu.dk/services/MLST/
